# Responses of Human Endothelial Cells to Pathogenic and Non-Pathogenic *Leptospira* Species

**DOI:** 10.1371/journal.pntd.0000918

**Published:** 2010-12-14

**Authors:** Denise G. Martinez-Lopez, Mark Fahey, Jenifer Coburn

**Affiliations:** 1 Tufts University School of Medicine, Boston, Massachusetts, United States of America; 2 Division of Infectious Diseases, Center for Infectious Disease Research, Medical College of Wisconsin, Milwaukee, Wisconsin, United States of America; Weill Medical College of Cornell University, United States of America

## Abstract

Leptospirosis is a widespread zoonotic infection that primarily affects residents of tropical regions, but causes infections in animals and humans in temperate regions as well. The agents of leptospirosis comprise several members of the genus *Leptospira*, which also includes non-pathogenic, saprophytic species. Leptospirosis can vary in severity from a mild, non-specific illness to severe disease that includes multi-organ failure and widespread endothelial damage and hemorrhage. To begin to investigate how pathogenic leptospires affect endothelial cells, we compared the responses of two endothelial cell lines to infection by pathogenic versus non-pathogenic leptospires. Microarray analyses suggested that pathogenic *L. interrogans* and non-pathogenic *L. biflexa* triggered changes in expression of genes whose products are involved in cellular architecture and interactions with the matrix, but that the changes were in opposite directions, with infection by *L. biflexa* primarily predicted to increase or maintain cell layer integrity, while *L. interrogans* lead primarily to changes predicted to disrupt cell layer integrity. Neither bacterial strain caused necrosis or apoptosis of the cells even after prolonged incubation. The pathogenic *L. interrogans*, however, did result in significant disruption of endothelial cell layers as assessed by microscopy and the ability of the bacteria to cross the cell layers. This disruption of endothelial layer integrity was abrogated by addition of the endothelial protective drug lisinopril at physiologically relevant concentrations. These results suggest that, through adhesion of *L. interrogans* to endothelial cells, the bacteria may disrupt endothelial barrier function, promoting dissemination of the bacteria and contributing to severe disease manifestations. In addition, supplementing antibiotic therapy with lisinopril or derivatives with endothelial protective activities may decrease the severity of leptospirosis.

## Author Summary

Leptospirosis is a widespread zoonotic infection that primarily affects residents of tropical regions, but is seen occasionally in temperate regions as well. Leptospirosis can vary in severity from a mild, non-specific illness to severe disease that includes multi-organ failure and widespread endothelial damage and hemorrhage. To investigate how pathogenic leptospires affect endothelial cells, we compared the responses of two endothelial cell lines to infection by pathogenic versus non-pathogenic leptospires. Our analyses suggested that pathogenic *L. interrogans* and non-pathogenic *L. biflexa* caused changes in expression of genes whose products are involved in cellular architecture and interactions with the matrix, but that the changes were in opposite directions, with infection by *L. biflexa* primarily maintaining cell layer integrity, while *L. interrogans* disrupted cell layers. In fact, *L. interrogans* caused significant disruption of endothelial cell layers, but this damage could be abrogated by the endothelial protective drug lisinopril. Our results suggest that *L. interrogans* binds to endothelial cells and disrupts endothelial barrier function, which may promote dissemination of the bacteria and contribute to severe disease manifestations. This disruption may be slowed by endothelial-protective drugs to decrease damage in leptospirosis.

## Introduction

Leptospirosis is a geographically widespread zoonosis that has emerged as a significant public health problem in urban slums, particularly in the tropics. The infection is caused by species of spirochetes belonging to the genus *Leptospira*. There are more than 200 serovars of *Leptospira* distributed among both pathogenic and non-pathogenic species [1]. The pathogenicity of different strains can vary considerably depending on the host species and age, and on the infecting serovar [2]. The spirochete's mode of entry is through mucous membranes and cuts or abrasions on the skin [1]. Upon entry, the organisms travel through the bloodstream to multiple sites, and may cause liver and kidney damage, meningitis, and a variety of other inflammatory conditions. If the host survives the acute infection, leptospires can persist in the proximal renal tubules for weeks to months, protected from antibodies and causing little to no inflammation. The bacteria are then shed in the urine, and animal urine contamination of water is the primary source of human exposure.

Although little is known about how *Leptospira* species establish infection in their hosts, adhesion to the host cell surface and extracellular matrix (ECM) by pathogens is often the first critical step in the initiation of infection. Several groups have investigated the adhesion of *Leptospira interrogans* to endothelial, fibroblast, kidney epithelial, and monocyte-macrophage cell lines cultured *in vitro* [3]–[9]. It is likely that pathogenic leptospires can attach to several different types of mammalian receptors to establish the infection. In fact, infectious strains of *Leptospira* have been shown to adhere to ECM components including collagen type IV, fibronectin and laminin, and also to the plasma protein fibrinogen [4], [10]–[12]. Adhesion to several ECM components is mediated at least in part by the LigA and LigB proteins [11] and a group of additional related proteins that were identified through homology to a laminin binding protein [10], [12].

Several studies have shown that the adhesion of pathogens to mammalian cells will provoke multiple changes in the physiology and/or gene expression of the host. The host-pathogen interactions that define a disease are clearly complex. Microarrays are a powerful tool to explore those host-pathogen interactions by analyzing the transcriptional profiles of host cells or pathogens. Although it has been documented that temperature and osmolarity alter leptospiral gene expression [13], [14], no previously published research has focused on the mammalian cell responses to the bacteria. To understand how human endothelial cells alter gene expression in response to incubation with different strains of *Leptospira*, human gene arrays were probed with cDNA derived from the RNA purified from infected cells and uninfected controls. In this study, we discuss how global analysis of gene expression allows us to gain insights into host specific responses to infection with pathogenic *Leptospira*.

## Materials and Methods

### Cell culture

The human microvascular endothelial cell line of dermal origin (HMEC-1) [15] was obtained from Dr. Ades (Centers for Disease Control and Prevention, Atlanta, Georgia) and cultured in endothelial basal medium (Clonetics, San Diego, CA) supplemented with 15% heat-inactivated fetal bovine serum (Hyclone, Logan, UT), 1 µg/ml hydrocortisone (Sigma-Aldrich, St. Louis, MO) and 10 ng/ml epidermal growth factor (Sigma-Aldrich). The immortalized human macrovascular endothelial cell line EA.hy926 [16] was kindly provided by Dr. C.-J. Edgell (University of North Carolina, Chapel Hill, NC) and grown in Dulbecco's modified Eagle medium with high glucose supplemented with 10% heat-inactivated fetal bovine serum (Gibco, Grand Island, NY) and HAT Media Supplement (Sigma-Aldrich). Both cell lines were cultured in the medium recommended by the supplier in a humidified atmosphere of 5% CO_2_ and both cell media were supplemented with 1 U/mL penicillin, 1 µg/mL streptomycin, and 2 mM L-glutamine for routine propagation. Cells to be used for experimental infection with *Leptospira* strains were cultured without the antibiotics.

The roles of proteoglycans in the endothelial cell response to *L. interrogans* were tested based on previously published protocols [17]. Briefly, chondroitin sulfate B was shown to bind *L. interrogans* and to competitively inhibit *L. interrogans* to mammalian cells, so it was tested for the ability to inhibit the endothelial cell responses to the bacteria described below. In addition, inhibition of proteoglycan synthesis by β-xyloside, which also decreases *L. interrogans* attachment to mammalian cells, was tested for any effect. Controls included chondroitin sulfate A, to which *L. interrogans* does not bind, and the sugar analog α-galactoside, which does not affect proteoglycan synthesis.

### Bacterial culture

The reference strain *Leptospira biflexa* serovar Patoc was obtained from the American Type Culture Collection (ATCC 23582, Manassas, VA), and is a non-pathogenic species. *L. interrogans* serovar Canicola (pathogenic, strain ATCC 23606 and strain 11203-32) were obtained from the ATCC and Dr. Richard Zuerner (USDA, Ames, IA), respectively. *L. interrogans* serovar Copenhageni (pathogenic, strain designated Fiocruz L1-130) was provided by Dr. David Haake (UCLA, Los Angeles, CA). Bacterial strains were maintained in ambient air at 30°C. Bacteria utilized for this study were at low passage from the suppliers (≤passage 6) and cultured in EMJH medium [1] supplemented with 100 µg/ml of 5-fluorouracil and 1% rabbit serum (Sigma-Aldrich). For some experiments, the bacteria were radiolabeled by addition of ^35^S cysteine plus methionine to the medium as described previously [17]. The bacteria were enumerated using a Petroff-Hausser counting chamber and dark field microscopy.

### Infection of endothelial cells for microarray analysis

Mammalian cells were plated in T-225 tissue culture flasks (BD Falcon, Bedford, MA) and grown up to 90% or higher confluence. When cells reached desired confluence, the monolayer was washed with PBS and the cells were lifted off the plastic culture flask with 5mM EDTA in PBS. This was done to allow access of the bacteria to endothelial cell surface receptors that are normally involved in attachment to the substratum, i.e. receptors that the bacteria may encounter when penetrating the vasculature. In addition, this approach minimizes degradation of mRNA that occurs during harvesting of adherent cells. After lifting, cells were spun for 10 minutes at 1,000 rpm, resuspended in the cell culture medium without antibiotics, and enumerated using a hemocytometer counting chamber. 2×10^7^ cells per sample were incubated in suspension with either *L. biflexa* serovar Patoc or *L. interrogans* serovar Canicola, or without any bacteria, for 1 h and 3 h at room temperature in the cell medium without antibiotics. The MOI (multiplicity of infection) used was 10 bacteria per mammalian cell. After incubation, cells were washed with phosphate buffered saline (PBS) and harvested for RNA isolation. The RNA was purified using RNeasy kit (Qiagen, Valencia, CA) with DNase digestion according to manufacturer's manual. The quality of RNA was checked using a Bioanalyzer (Agilent, Santa Clara, CA).

### Microarray analysis

Human HEEBO (Human Exonic Evidence Based Oligonucleotide) Arrays, consisting of 44,544 70mer probes representing 30,718 known genes, were purchased from Microarrays Inc. (Nashville, TN). 5 to 20 µg of total RNA from uninfected control and infected samples was used to generate cDNA labeled with aminoallyl (aa)-dUTP through a reverse transcription reaction using anchored oligo(dT) primers. The purified aa-dUTP-labeled cDNAs were coupled in 10 µl 0.1 M NaHCO_3_ with either Cy3 or Cy5 NHS-ester dye. Cy-dye labeled cDNA was purified using a Cyscribe GFX column (Amersham Biosciences, Piscataway, NJ). The two differently labeled cDNAs were mixed and hybridized using Pronto Microarray Hybridization Kit in a hybridization chamber (Corning, Corning, NY), with the same array slide for 38 to 42 hr according to manufacturer's instruction. After a series of washes using the buffers provided in the kit, slides were spun dry and scanned under two laser channels in a Scanarray 4000 scanner (Packard Bioscience, Meriden, CT).

Images were overlaid and analyzed using Imagene (BioDiscovery, El Segundo, CA). Raw gene expression was imported from Imagene to GeneSifter (GeneSifter.Net, VizX Labs, Seattle, WA) for analysis. Data from 3 biological replicate experiments were normalized using Lowess normalization and by the median of the raw intensities for all spots in each sample for each array. The ratio of two fluorescence intensities of each spot reflected the ratio of each gene expressed in the infected and uninfected samples. Genes were considered to be induced or repressed when the ratio of infected/uninfected was at least 1.5 fold (increased or decreased), and the P value was <0.05 by the Student's two-tailed *t* test. For analysis involving more than one time point and/or condition, the one way ANOVA test was performed. Microarray data are deposited in GEO archive under the accession numbers GSE23172 and GSE23173.

### Fluorescence microscopy

EA.hy926 cells were seeded in tissue culture treated glass slides (BD Falcon) and grown at 37°C as described above. After cells reached 100% confluence, the monolayer was washed three times with PBS and medium without antibiotics was added. Four compartments of each slide were inoculated with 1×10^7^ bacteria (MOI = 10) of either *L. biflexa* serovar Patoc or *L. interrogans* serovar Canicola. The remaining four wells were left uninfected to serve as negative controls. In some cases, parallel experiments were performed using cells plated on coverslips in 24 well culture dishes, which allowed centrifugation to facilitate bacterial-endothelial cell contact. At the end of the incubation (1 h, 3 h and 24 h) the slides were washed three times with PBS and fixed with 3% (wt/vol) paraformaldehyde in PBS at room temperature for 30 min. Cells were permeabilized with 0.1% Triton X-100 in PBS, washed three more times with PBS, and blocked overnight at 4°C with HEPES buffered saline (HBS) and 1% bovine serum albumin (BSA). On the next day the slides were washed again with PBS and incubated with fresh blocking solution for 1h at room temperature. After blocking, the layers were probed with either rabbit anti-*L. interrogans* (a gift from Dr. Richard Zuerner, USDA, AMES, IA) diluted 1∶5000 or anti-*L. biflexa* antiserum (Biogenesis, Inc., Brentwood, NH) diluted 1∶1000, followed by anti-rabbit IgG-TRITC conjugate (1∶1000) plus phalloidin-FITC (200 U/mL) to stain filamentous actin. After repeated washing in PBS, chambers were removed from the slides and Prolong Anti-Fade (Invitrogen, Carlsbad, CA) was used to mount coverslips. Two different microscopes at two different institutions were used throughout the course of this work. At institution one, images were captured using a Zeiss Axioplan microscope with a digital charge-coupled device camera (Hamamatsu, Hamamatsu City, Japan) and co-localization of the fluorescent labels was done using Volocity software (Improvision Inc., Lexington, MA). At the second institution a Zeiss Axioimager Z1 with an Axiocam HrC camera and a Nuance Multi-Spectral Imaging System (software CRI Inc, Woburn, MA, v.2.6.0) was used.

### Transendothelial migration assay

The endothelial cell lines EA.hy926 and HMEC were plated in 3.0 µm (2×10^6^ pores/cm^2^) polyester transwell inserts (Corning) and cultured as described above. After reaching 100% confluence, as assessed by lack of penetration of the fluorescent dye FITC-dextran 40,000 (and loss of penetration of the *L. biflexa* serovar Patoc), the monolayer was washed with PBS and cell medium without antibiotics was added to the inserts and wells. Inserts without cells were used as controls for these experiments. Bacteria were added to an MOI of 50 to allow reliable enumeration of bacteria crossing the cell layers or membranes without cells at early time points, and 10 µL from the insert and from the well were taken after 1 h, 3 h, 6 h, 24 h, 27 h, 48 h and 72 h. In addition to the non-pathogenic strain Patoc and the pathogenic Canicola, *Leptospira interrogans* serovar Copenhageni was also used to analyze the migration of leptospires through the cell monolayer. Motile leptospires were counted by dark-field microscopy using a Petroff-Hausser chamber. Data are shown for the time points through which the bacteria were motile; after 72 hr there was a progressive decrease in *L. biflexa* motility.

### Assessment of endothelial cell viability

To determine whether the bacteria were affecting the viability of the endothelial cells, four methods were used. First, adherent and EDTA-lifted endothelial cells infected at an MOI of 10 were washed, then incubated with the vital dye CellTracker Green (CT-CMFDA, 10 µM) plus DAPI (0.02 µg/ml) (Molecular Probes, now part of Invitrogen, Eugene, OR) for 1 hour at 37°C under 5% CO_2_. The samples were mounted and viewed using the Zeiss Axioplan microscope described above, and live cells (bright green cytoplasm) and dead cells (bright blue nuclei) were enumerated in at least three fields per sample in at least three independent experiments. Second, the cells were stained using the Vybrant Apoptosis Assay Kit 2 (Molecular Probes), which stains for annexin V and membrane permeability. Third, the APO-BrdU TUNEL kit, also from Molecular Probes, was used. A second TUNEL-based kit, Alert DNA Fragmentation kit (Clontech Laboratories, Inc., Mountain View, CA) was also used. For methods two and three, the cells were also assessed using fluorescence microscopy. Finally, cells were harvested, and DNA was purified and analyzed for fragmentation (an assessment of apoptosis) using conventional agarose gel electrophoresis.

## Results and Discussion

We identified statistically significant and reproducible changes in endothelial cell gene expression after incubation with each bacterial strain as compared to the uninfected controls and to each other. The data were analyzed using Webgestalt [18] to identify mammalian cell genes whose products comprise functional pathways in which multiple components showed alterations in gene expression (Table 1). Four pathways that show internally consistent changes in gene expression are the KEGG focal adhesion, regulation of actin cytoskeleton, leukocyte transendothelial migration, and ECM-receptor interaction pathways. They are considered together because a number of genes encode proteins whose functions participate in aspects of cell biology common to these pathways.

**Table 1:** Clusters of ≥3 genes with statistically significant changes in expression common to Ea.hy926 endothelial cells infected with *L. biflexa* serovar Patoc and *L. interrogans* serovar Canicola.

KEGG pathway^1^	genes, *L. biflexa*	p value, *L. biflexa* 2	genes, *L. interrogans*	p value, *L. interrogans*
Focal adhesion (FA)	7	1.00e-6	7	7.94e-6
Regulation of actin cytoskeleton (ACT)	3	1.75e-2	4	6.59e-3
Leukocyte transendothelial migration (TEM)	2	3.83e-2	3	8.73e-3
Phosphatidylinositol signaling system (PI)	3	9.28e-4	2	2.78e-2
ECM-receptor interaction (ECM)	3	1.53e-3	2	3.82e-2

1 KEGG Pathways: http://www.genome.jp/kegg/pathway.html. The Table is arranged in order of descending p value for *L. interrogans*. The comparisons were made between cells infected with the indicated bacterial strain vs. uninfected controls at 1 hr. post infection. Similar trends were observed at the 3 hr. time point. Pathway abbreviations are provided for reference to Table 2. The same pathways also showed significant changes in expression, in similar patterns, in a second endothelial cell line, HMEC (data not shown).

2 P values considered to be statistically significant were <0.05 by the Hypergeometric test. *(* http://bioinfo.vanderbilt.edu/webgestalt *)*.

Actin microfilaments are one of the three major components of the cellular cytoskeleton. The cytoskeleton participates in maintaining adhesion to and communicating with the extracellular matrix, cell migration, division, and signaling. β-Actin (ACTB) mRNA was decreased in response to *L. interrogans* but increased in response to *L. biflexa*, both as compared to the uninfected control cells (Table 2). Guanine nucleotide-binding protein alpha-13 subunit (GNA13) mediates the activation of the small GTPase RhoA [19] which when activated controls the assembly of focal adhesions and actin in the formation of stress fibers [20]. Although RhoA was not differentially regulated in response to the bacteria, Rho GTPase activating protein 5 (RhoGAP5) was differentially expressed following the same pattern as GNA13, in which both genes were downregulated in response to the pathogenic leptospires in comparison to the uninfected controls, and upregulated in response to the non-pathogen. The effect of decreased GNA13 may be to decrease stimulation of Rho, while decreasing the GAP would decrease inactivation of Rho with concomitant decreased cell spreading on the extracellular matrix.

**Table 2:** Ea.hy926 endothelial cell genes affected differently by *L. biflexa* Patoc vs. *L. interrogans* Canicola infection.

Gene (KEGG Pathway(s))	Product	Fold change in cells infected with *L. biflexa* vs. uninfected	Fold change in cells infected with *L. interrogans* vs. uninfected	Predicted functional significance
RDX (ACT)	radixin	+2.01	−3.08	Less linkage of actin cytoskeleton to cell membrane in cells infected with *L. interrogans* vs. *L. biflexa* (reviewed in [48])
LAMB1 (ECM, FA, ACT)	laminin β1	+1.84	−2.80	Less synthesis of this ECM component in cells infected with *L. interrogans* vs. *L. biflexa*
CAV1 (ECM, FA)	caveolin 1	+1.67	−2.48	*L. interrogans vs. L. biflexa*: decreased MAPK signaling through integrins, expected as cells lose attachment to substrate (reviewed in [49])
ITGAV (FA, ECM, ACT)	integrin αv	+1.69	−2.46	Less adhesion to ECM in cells infected with *L. interrogans* vs. *L. biflexa* (reviewed in [50])
CAV2 (ECM, FA)	caveolin 2	+1.81	−2.30	*L. interrogans* vs. *L. biflexa*: decreased MAPK signaling through integrins, expected as cells lose attachment to substrate (reviewed in [49])
NCKAP1 (ACT)	NCK-associated protein 1	+1.59	−2.44	Decreased actin remodeling in lamellipodia in cells infected with *L. interrogans* vs. *L. biflexa* [51]
PIP5K1A (PI, FA, ECM, ACT)	Phosphatidyl-inositol-4-phosphate 5-kinase type I alpha	+1.52	−2.50	less focal adhesion in cells infected with *L. interrogans* vs. *L. biflexa* [52]
ARHGAP5 (ACT, FA, TEM)	RhoGAP5 (p190 RhoGAP)	+1.66	−2.27	less inactivation of Rho GTPase, less cell spreading, in cells infected with *L. interrogans* vs. *L. biflexa* (reviewed in [53])
ACTB (ACT, FA, TEM)	β-actin	+1.95	−1.83	decreased actin levels in cells infected with *L. interrogans* vs. *L. biflexa*
GNA13 (ACT)	G protein alpha 13	+1.51	−1.81	less activation of Rho GTPases in cells infected with *L. interrogans* vs. *L. biflexa* [19]
TTN (ECM)	titin (connectin)	+1.78	−1.78	decreased actin polymerization, or rates thereof, in cells infected with *L. interrogans* vs. *L. biflexa* [54]
TNC (ECM, ACT)	tenascin	−1.66	+1.90	more synthesis of this ECM component in cells infected with *L. interrogans* vs. *L. biflexa*
ITGB1 (FA, ECM, ACT, TEM)	integrin β1	+1.54	−1.79	less adhesion to ECM in cells infected with *L. interrogans* vs. *L. biflexa* (reviewed in [50])

The Table is arranged in order of descending differences between *L. interrogans* vs. *L. biflexa*-infected Ea.hy926 cells in comparison to the uninfected controls at I hr post-infection. For each gene, relevant KEGG pathway abbreviations are noted (see Table 1).

The changes in expression of several additional genes are consistent with changes in cellular architecture as a result of leptospiral infection of these endothelial cells. For example, decreases in the mRNAs for radixin (RDX, a protein that links the actin cytoskeleton to the plasma), caveolins 1 and 2 (CAV1 and CAV2, which couple integrins to the Ras-ERK pathway, titin, the ECM component laminin β1, and integrin subunits α_v_ and β_3_ (Table 2), were seen in cells infected with *L. interrogans* Canicola as compared to the uninfected controls. In contrast, the *L. biflexa* Patoc caused increases in mRNA levels for the same genes in infected cells vs. uninfected controls (Table 2). Together, all of these gene expression patterns are consistent with the hypothesis that one effect of *L. interrogans* serovar Canicola is to promote actin remodeling and detachment of the cells from the ECM. A fundamental stage in the pathogenesis of *Leptospira* infections is the ability of the bacteria to cross mucous membranes and underlying epithelial barriers, as well as endothelial cell barriers, and disseminate to different organs. Although *Leptospira* species are extracellular bacteria apparently devoid of actin modifying exotoxins [21]–[23], and devoid of the specialized secretion systems utilized by many bacterial pathogens to deliver toxins that disrupt the host cell cytoskeleton (as reviewed in [24]–[28]), pathogenic leptospires might be indirectly targeting the cytoskeleton *via* cell surface attachment mechanisms that co-opt the host cell signaling to achieve the same result.

**Figure 1:**
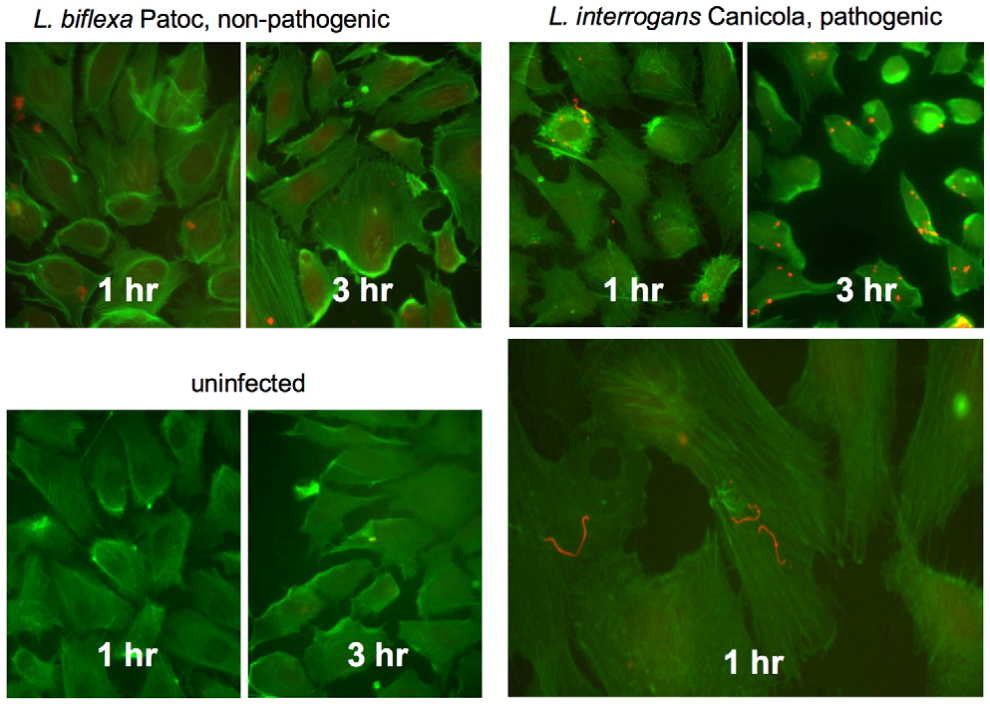
*L. interrogans* causes disruptions in endothelial cell monolayers. Ea.hy926 endothelial cells were plated in tissue culture treated glass chamber slides and allowed to reach near confluence (assessed visually). The bacteria were added at MOI = 10 and incubated with the endothelial cells for 1 or 3 hours at 37°C, then were washed and fixed. The slides were stained with phalloidin-FITC, which illuminates F actin, plus anti-*Leptospira* antibodies followed by TRITC- conjugated secondary antibody. Retraction of the cell bodies in response to *L. interrogans* Canicola, but not *L. biflexa* Patoc, is evident, particularly at 3 hr infection. The brighter staining of rounded and retracted cells with FITC-phalloidin may be due to disorganization of cellular architecture without complete depolymerization of the actin, which in the increased depth and decreased area of the cytoplasm would appear more concentrated and therefore brighter. Changes in endothelial cell morphology were most evident, and at earlier time points, in cells with which the *L. interrogans* bacteria were associated. One higher magnification micrograph of *L. interrogans* Canicola infected cells is included because the bacteria are small when viewing fields of endothelial cells that provide information on integrity of the monolayer. Micrographs are representative of multiple (>12) independent experiments. *L. interrogans* Copenhageni caused essentially the same changes in endothelial cell morphology as *L. interrogans* Canicola (data not shown).

Decreased cellular adhesion to the ECM and rearrangement of the cytoskeleton may facilitate the migration of *Leptospira* through endothelial barriers as it disseminates from the site of inoculation. To further explore the possibility that actin rearrangements are triggered by *Leptospira* infection at the functional level, endothelial cells plated in chamber slides were infected at an MOI of 10 for 1 hour and 3 hours. As shown in Figure 1, the bacteria were clearly more adherent to the cells than to the extracellular space, and the pathogenic bacteria caused dramatically more significant alterations in cellular morphology and integrity of the cell layer than did the non-pathogenic bacteria. The earliest change noted was a reduction in cortical actin (so the cell edges are less defined) and appearance of gaps in confluent cell layers, followed by loss of stress fibers and rounding of the cells. The images shown in Figure 1 are from cell layers that were just below confluence prior to infection, to allow better visualization of changes in individual cells. For example, while the cortical actin has largely disappeared in cells infected with *L. interrogans* Canicola by 1 hour post-infection, and stress fibers have disappeared and cell rounding is evident by 3 hours, the cells are largely unaffected at the same time points after infection with *L. biflexa* Patoc (Figure 1). *L. biflexa* does adhere to mammalian cells in culture less efficiently than does *L. interrogans* (as shown and reviewed in [17]), but even when bacterial contact with the cells was facilitated by centrifugation, the *L. biflexa* caused little disruption to cellular morphology and cell layer integrity (data not shown).

**Figure 2:**
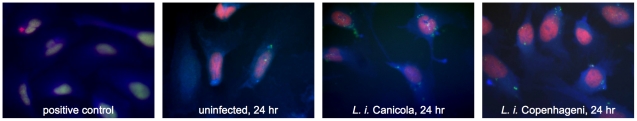
*L. interrogans* infection does not trigger apoptosis in endothelial cells. Ea.hy926 cells were infected with *L. interrogans* Canicola at MOI = 10 for 24 hr, then stained with the TUNEL-based ApoAlert DNA fragmentation kit (Clontech Laboratories, Inc.) according to the manufacturer's instructions. As a positive control, the cells were permeabilized, then treated with DNase1 at 1 µg/ml for 10 min at ambient temperature prior to staining. TUNEL staining (green nuclei) indicates DNA fragmentation consistent with apoptosis. Nuclei are also stained with propidium iodide (red), and the F actin cytoskeleton is stained with phalloidin (blue). The micrographs do not demonstrate any indication of apoptosis in *L. interrogans*-infected cells, and are representative of multiple experiments using multiple different tests for apoptosis, each performed at least three times.

**Figure 3:**
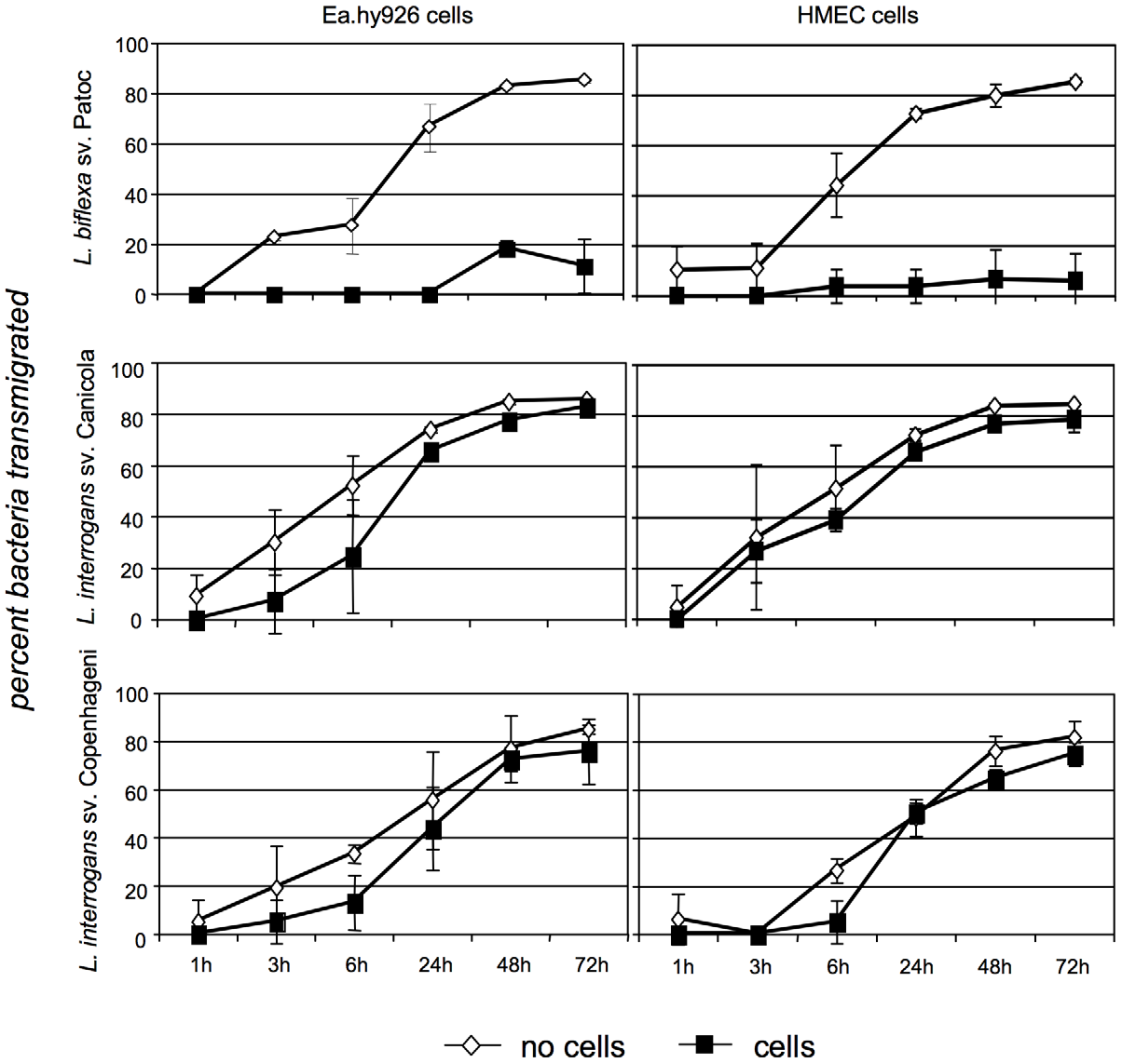
*L. interrogans* transmigrates across endothelial cell monolayers more efficiently than does *L. biflexa*. Ea.hy926 and HMEC endothelial cells were placed in 3 µm transwell inserts (“upper chambers”) in 24 well dishes containing medium and allowed to reach complete confluence (assessed visually and with FITC-dextran 40,000). The bacteria were added at MOI = 50 (providing sufficient numbers for quantification) to wells with and without endothelial cells, and samples were taken for counting from both chambers of the transwell plates at the times indicated. The graphs show bacteria that have migrated from upper to lower chamber (i.e. transmigration) as the percent of the total in both the upper and lower chambers. Shown are the means ± standard deviations of 3 independent experiments. For *L. interrogans* sv. Copenhageni and Canicola, the rates of transmigration through membranes with and without cells were not statistically significant as assessed by repeated measures ANOVA followed by Bonferroni's multiple comparison test (P>0.05). For *L. biflexa*, the same comparison was significantly different, P<0.001. In addition, the *L. interrogans* strains were significantly different from *L. biflexa* in crossing the cell layers (P<0.05).

Although these and subsequent experiments were performed using adherent cells, the morphologic changes are consistent with changes in mRNA levels seen using lifted cells in the microarray experiments. Despite the alterations in cellular architecture and monolayer integrity, no decrease in endothelial cell viability was found by any of several criteria (see Materials and Methods), even after infection times extended as long as 48 hours (Figure 2). The disruptions in the layers did, however, result in the ability of the pathogenic strain to cross the monolayers more efficiently than did the non-pathogenic bacteria (Figure 3). After a brief period in which the endothelial layer did prevent significant transmigration of the bacteria, the layer rapidly became essentially irrelevant as a barrier to the penetration of the pathogenic bacteria, as the bacterial counts in the lower chamber were unaffected by whether or not cells had been plated on the membrane.

**Figure 4:**
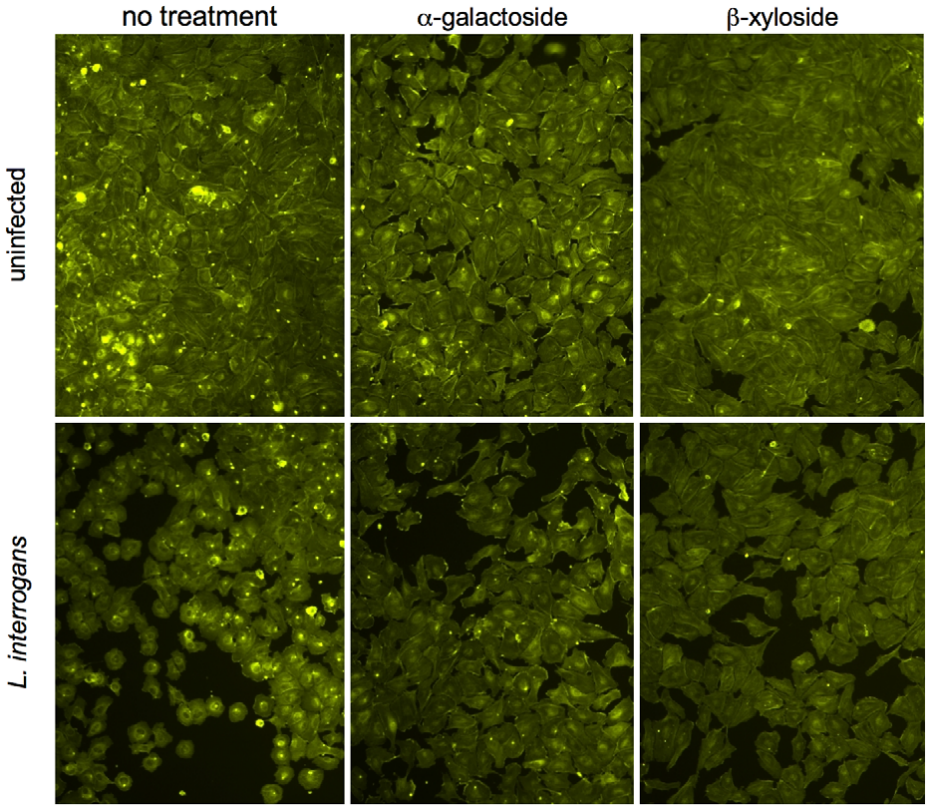
Interference with proteoglycan binding by *L. interrogans* does not prevent disruption of endothelial cell layers in culture. Ea.hy926 cell layers were treated with the proteoglycan synthesis inhibitor β-xyloside, or the control α-galactoside, as described in [17] prior to infection with *L. interrogans* sv. Copenhageni. After 3 hr, the cell layers were washed and fixed, then stained with phalloidin-FITC. Neither reagent significantly reduced disruption of the cell layers by *L. interrogans*, as alterations in cell morphology and significant gaps between cells were seen when *L. interrogans* was present, and trans-endothelial cell layer migration was not significantly affected (data not shown). Consistent with this result, chondroitin sulfates B and A, which do and do not inhibit *L. interrogans* attachment to mammalian cells, respectively [17], also had no effect (not shown).

Because *Leptospira interrogans* has been shown to bind to proteoglycans on the mammalian cell surface [17], we tested a proteoglycan synthesis inhibitor, β-xyloside, for the ability to decrease damage to endothelial cell layers caused by *L. interrogans* Canicola. β-xyloside inhibits transfer of glycosaminoglycan chains to protein cores; a control sugar analog, α-galactoside, was tested in parallel. As shown in Figure 4, inhibition of proteoglycan synthesis did not fully prevent the damage to the endothelial cell layers caused by *L. interrogans*. The inhibition of glycosaminoglycan chain attachment does not significantly affect the formation of holes in the cell layer caused by *L. interrogans* Canicola as assessed visually and by measurement of *L. interrogans* penetration of the cell layers (data not shown). β-xyloside does cause a reduction of *L. interrogans* Canicola and Copenhageni attachment to these cells ([17] and data not shown), but does not abolish bacterial attachment, consistent with the hypothesis that additional non-proteoglycan molecules serve as substrates for *L. interrogans* attachment to cells. Direct bacterial attachment to the cells does appear to be required for the damage to the endothelial cell layers, as supernatants harvested from infected cell layers (infection times of 1–24 hr) and sterilized by centrifugation and filtration through 0.1 µm filters did not affect endothelial cell layer integrity (data not shown). Therefore, non-proteoglycan cell surface receptors are likely to be those primarily involved in the responses of the endothelial cells to *L. interrogans* attachment, and efforts to identify both the host cell and the bacterial cell molecules involved in these interactions are underway. As noted in the publication reporting the sequence of two *L. biflexa* Patoc strains [29], there are a number of proteins predicted in the published *L. interrogans* genomes that are not present in the *L. biflexa* Patoc genome, including some that are postulated to have potential adhesin activities. These include proteins containing leucine-rich repeats, which are involved in many protein-protein interactions [29]. As stated in the publication of the *L. biflexa* genome, it is intriguing that a *Treponema denticola* leucine-rich repeat protein, LrrA, has been identified as an adhesion/tissue penetration factor [29], [30]. It is also possible that additional components of the surfaces of *L. interrogans* and *L. biflexa* might have different effects on host cells [31]–[33]. At this point, however, the determinants critical to the effects of *L. interrogans*-host cell interaction reported here remain to be identified, and neither bacterial adhesins nor host substrates can necessarily be predicted solely on the basis of the primary amino acid sequences.

**Figure 5:**
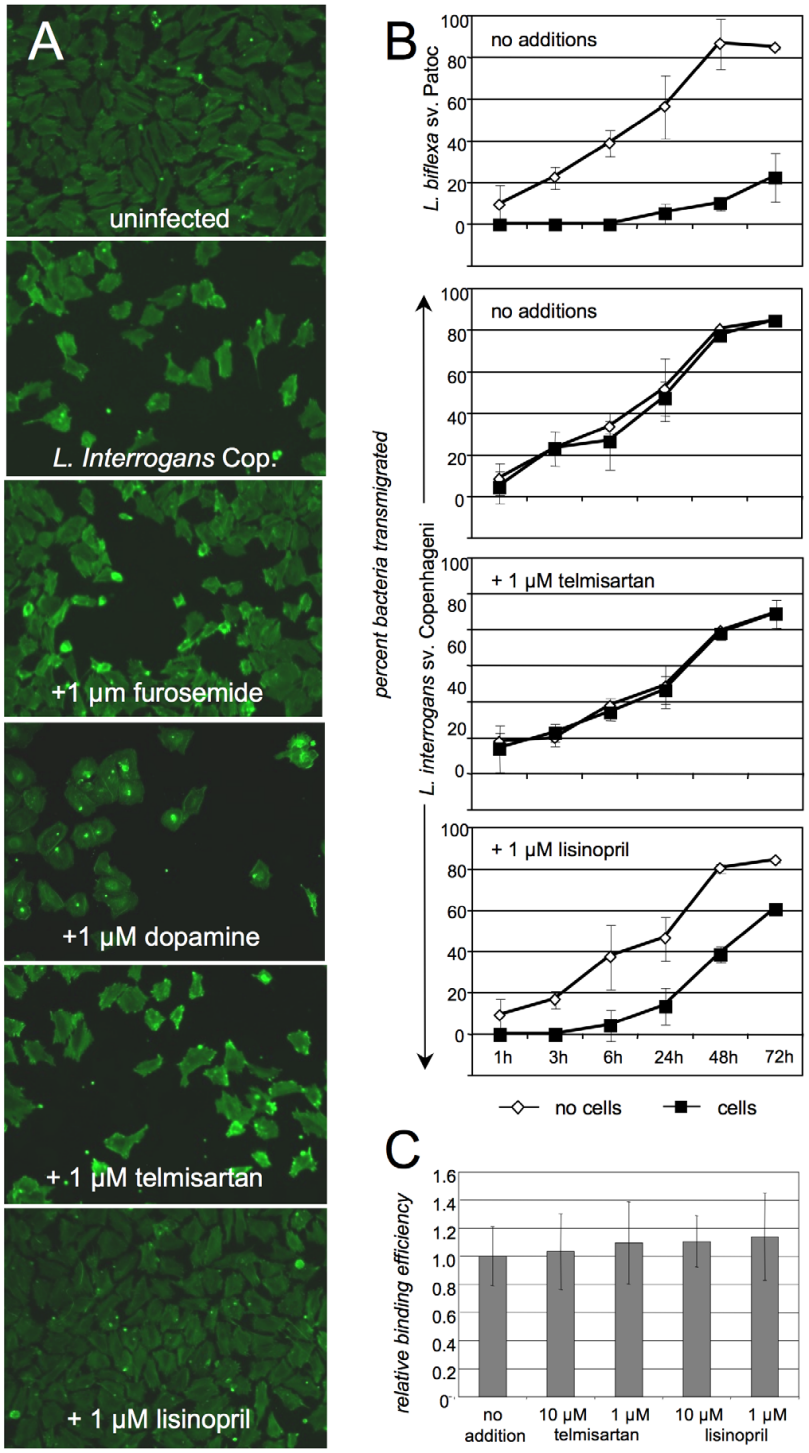
Effects of specific drugs that protect endothelial barrier function on damage caused by *L. interrogans*. Panels A and B: Ea.hy926 endothelial cell layers were infected with *L. interrogans* sv. Copenhageni or *L. biflexa* sv. Patoc as described in Materials and Methods, except that just prior to the addition of the bacteria the drugs lisinopril, telmisartan, dopamine, or furosemide were added to 1 µM. The micrographs shown in Panel A (representative of three experiments) were taken at the 6 hour time point; the graphs in Panel B show the transmigration of leptospires over the entire 72 hr. time course. Shown are the means and standard deviations of all data from three experiments. Statistical significance was determined using repeated measures ANOVA followed by Bonferroni's multiple comparison test. For wells with cells, *L. interrogans* vs. *L. biflexa*, p<0.001, *L. interrogans* with no additions vs. telmisartan p>0.05 (not significant), *L. interrogans* with no additions vs. lisinopril p<0.001, *L. interrogans* with telmisartan vs. lisinopril p<0.001. There were no significant differences in the absence of cells, and the drugs did not affect bacterial motility or attachment of ^35^S-labeled leptospires to the cells (Panel C and data not shown).

Several drugs currently in use in humans have been reported to have endothelial barrier protective function; all are in use as anti-hypertensive therapeutics, and some for other therapeutic purposes as well. We therefore tested four different drugs with different mechanisms of action for the ability to prevent the damage to endothelial layers in culture caused by *L. interrogans*. Lisinopril binds to and competitively inhibits angiotensin 1 binding to angiotensin converting enzyme (ACE), which is expressed by endothelial cells, while telmisartan competitively inhibits angiotensin 2 binding to its receptor AT_1_. Dopamine is an antagonist of VEGF/VEGFR2-mediated cell layer permeability in treatment of human umbilical vein endothelial cells (HUVECs) *in vitro* at 10µM, as well as VEGF-mediated angiogenesis *in vivo* and proliferation of HUVECs at 1 µM *in vitro* [34], [35]. Furosemide is an anion transport blocker and is used as a diuretic but has anti-hypertensive activity as a consequence, and was used as a control not expected to preserve endothelial layer integrity. While telmisartan, furosemide, and dopamine did not protect the endothelial layers from the damage due to *L. interrogans* Copenhageni infection, lisinopril did at 100 nM, 1 µM and 10 µM (Figure 5, representing 3 independent experiments, and data not shown). There are several possible explanations for this, including: 1) lisinopril inhibits *L. interrogans* attachment to the cells, and 2) that attachment is unaffected but the interaction of the bacteria triggers activation of a signaling cascade or release of a mediator whose action or activation is inhibited by lisinopril. We therefore investigated the possibility that lisinopril might prevent endothelial damage by blocking *L. interrogans* Copenhageni attachment to the cells, but no inhibition of adhesion of ^35^S-labeled bacteria [17] was seen even at a concentration of lisinopril 10 fold over the concentration used for these experiments (Figure 5).

**Figure 6:**
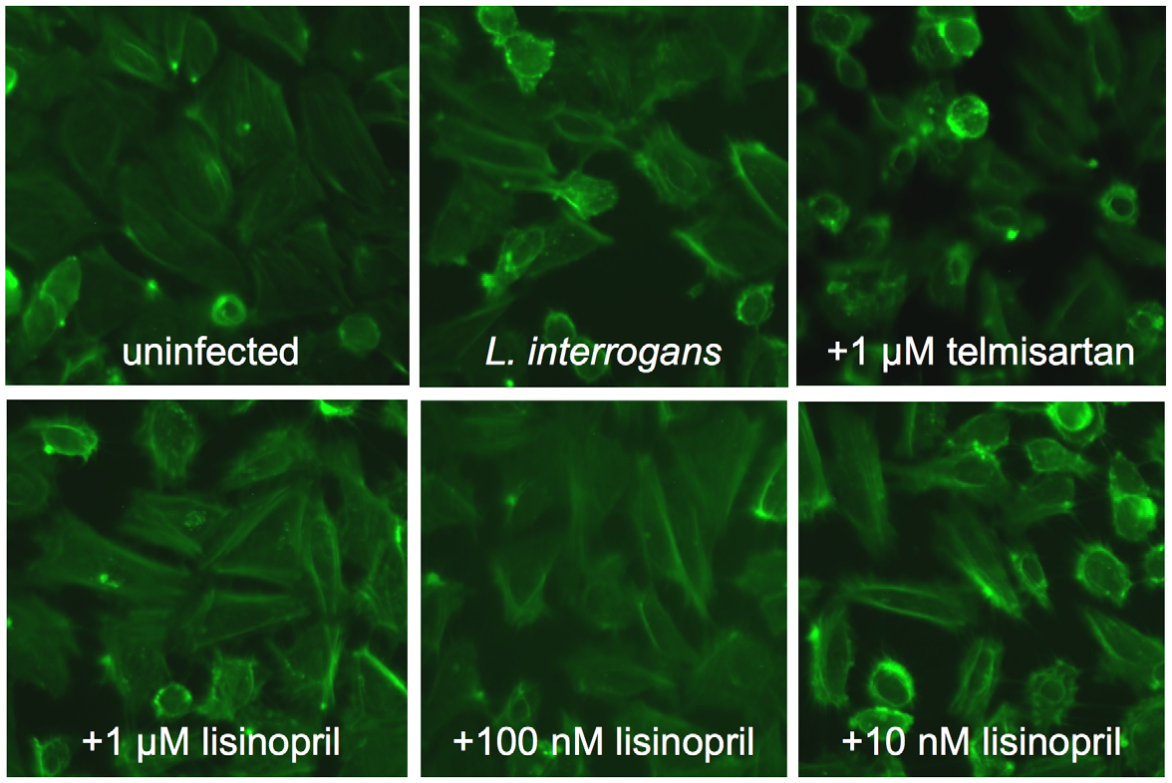
Lisinopril concentrations effective in protection of endothelial cell layers from damage due to *L. interrogans*. Ea.hy926 endothelial cell layers were infected with *L. interrogans* sv. Copenhageni as described in Materials and Methods, except that just prior to the addition of the bacteria the drugs lisinopril or telmisartan were added to the concentrations indicated. The micrographs were taken at the 3 hour time point. Lisinopril at 10 µM, 1 µM and 100 nM blocked endothelial disruption by *L. interrogans*; lisinopril at 10 nM or below did not.

Although it was tempting to speculate that cell-surface-localized ACE could serve as a receptor for *L. interrogans*, as the enzyme is expressed by endothelial cells and proximal tubule epithelial cells [36], and is therefore open to possible competition by the lisinopril, this is not consistent with our results to date. However, ACE2 is not inhibitable by lisinopril, but is a receptor for the SARS virus [37], so there is precedent for ACE proteins serving as receptors for pathogens. It is also possible that the effect of lisinopril in our system is not related to ACE inhibition, but is instead due to additional effects of lisinopril, such as inhibition of isoprenoid synthesis, which is required for the post-translational modification of Rho GTPases, which in turn regulate the actin cytoskeleton [38]. In turn, this may lead to increased NO synthesis, which is protective of endothelial function in the face of a variety of insults. Given that doxycycline also has endothelial protective effects [39], and that doxycycline is effective in treating leptospirosis [40], our results may also provide a starting point for investigation into possible combinatorial therapeutic approaches to reduction of endothelial damage and consequent organ damage in human populations during leptospirosis outbreaks. Should this combinatorial approach prove useful in animal models, consideration as a focused approach to the treatment of human leptospirosis is warranted. The 1 µM dose shown in Figure 5 is at the high end of the physiologically relevant dosing range for humans, but administration of an antihypertensive to a patient with clinical manifestations of leptospirosis would be contraindicated, as further depression of blood pressure levels would be potentially lethal. However, in outbreak situations, this agent could potentially help to reduce endothelial damage if administered to affected populations as soon as an outbreak situation is recognized, prior to exposure of the majority of the population to pathogenic *Leptospira* species. In addition, protective effects of lisinopril were maintained even at a dose of 100 nM, which is well within the range routinely used in humans (Figure 6). It will also be interesting to investigate the possibility that, on a population basis, patients on lisinopril fare better than patients not on this therapy during leptospirosis outbreaks.

Reorganization of the actin cytoskeleton, as indicated by our microarray studies and by phalloidin staining of F actin, is essential to the pathogenesis of diverse bacterial infections, and pathogens use many different strategies to provoke changes in the cellular cytoskeleton in order to facilitate invasion of tissues, invasion of host cells, or evasion of phagocytosis (as reviewed in [24], [41], [42]). A different spirochete, *Treponema denticola*, produces the protein Msp, which disrupts the actin cytoskeleton in neutrophils and fibroblasts, preventing phagocytosis of the bacterium and inhibiting the cellular migration required to respond to and repair the damage caused by the pathogen and the host response at the site of infection [43], [44]. These activities are likely to facilitate invasion and colonization of periodontal tissues by *T. denticola*. Previous work by another laboratory demonstrated that *L. interrogans* Copenhageni crosses MDCK canine kidney epithelial cell layers in culture more rapidly than does *L. biflexa* Patoc [45], but without significant disruption to the cell layers or the actin cytoskeleton. Consistent with these results, in experiments not shown here we also observed no significant damage to NRK (normal rat kidney) 293 (human kidney) or HEp-2 (human laryngeal) epithelial cell layers infected with *L. interrogans* Canicola or *L. interrogans* Copenhageni. The calculations of the proportions of bacteria crossing the cell layers differed between the two studies, but our protocol accounted for the replication of the *L. interrogans* Canicola and Copenhageni in the co-cultures, while the *L. biflexa* Patoc did not replicate (data not shown). Thus the endothelial cells tested here respond very differently to the bacteria than did the MDCK epithelial cells, and our results are the first to suggest a mechanism: disruption of actin dynamics by bacterial attachment to the cell surface. Thus, while *L. interrogans* has not been shown to secrete a toxin that modifies actin, the bacteria are able to manipulate the actin cytoskeleton indirectly. Even the pore forming toxin activity reported for *Leptospira* [46], [47] does not appear to have as large an effect, as the endothelial cells here were viable throughout the experiments. The leptospires may be able to establish disseminated infection in part due to the binding of the bacteria to one or more mammalian cell surface receptors that in turn, regulate the dynamics of the actin cytoskeleton in the mammalian cell. Deciphering the role of, and mechanisms behind, actin rearrangement in response to pathogenic *Leptospira* will provide insights into the mechanisms that leptospires uses to disseminate to different organs of the host to cause infection and disease, and provides a possible avenue for therapeutic intervention in conjunction with antimicrobial therapy.
